# Characterization of a Broadly Neutralizing Monoclonal Antibody against SARS-CoV-2 Variants

**DOI:** 10.3390/v14020230

**Published:** 2022-01-24

**Authors:** Tasnim Saifudin Zakir, Tao Meng, Lee Ching Pei Carmen, Justin Jang Hann Chu, Raymond Tzer Pin Lin, Mookkan Prabakaran

**Affiliations:** 1 Temasek Life Sciences Laboratory, 1 Research Link, National University of Singapore, Singapore 117604, Singapore; tasnim@tll.org.sg (T.S.Z.); mengtao@tll.org.sg (T.M.); carmen@tll.org.sg (L.C.P.C.); 2 Translational Research Programme, Department of Microbiology and Immunology and Infectious Disease, Yong Loo Lin School of Medicine, National University of Singapore, Singapore 117604, Singapore; miccjh@nus.edu.sg (J.J.H.C.); raymond_lin@ncid.sg (R.T.P.L.); 3 Biosafety Level 3 Core Facility, Yong Loo Lin School of Medicine, National University of Singapore, Singapore 117599, Singapore; 4 National Public Health Laboratory, National Centre for Infectious Diseases, Singapore 308442, Singapore

**Keywords:** SARS-CoV-2 variants, spike RBD, neutralizing epitope, escape mutant, cross-neutralizing antibody

## Abstract

The constant mutation of SARS-CoV-2 has led to the emergence of new variants, which call for urgent effective therapeutic interventions. The trimeric spike (S) protein of SARS-CoV-2 is highly immunogenic with the receptor-binding domain (RBD) that binds first to the cellular receptor angiotensin-converting enzyme 2 (ACE2) and is therefore the target of many neutralizing antibodies. In this study, we characterized a broadly neutralizing monoclonal antibody (mAb) 9G8, which shows potent neutralization against the authentic SARS-CoV-2 wild-type (WT), Alpha (B.1.1.7), and Delta (1.617.2) viruses. Furthermore, mAb 9G8 also displayed a prominent neutralizing efficacy in the SARS-CoV-2 surrogate virus neutralization test (sVNT) against the Epsilon (B.1.429/7), Kappa (B.1.617.1), Gamma (P.1), Beta (B.1.351), and Delta Plus (1.617.2.1) RBD variants in addition to the variants mentioned above. Based on our in vitro escape mutant studies, we proved that the mutations V483F and Y489H within the RBD were involved in ACE2 binding and caused the neutralizing evasion of the virus from mAb 9G8. The development of such a cross-reactive neutralizing antibody against majority of the SARS-CoV-2 variants provides an important insight into pursuing future therapeutic agents for the prevention and treatment of COVID-19.

## 1. Introduction

The continuous spread and rapid emergence of SARS-CoV-2 variants pose the greatest challenge to public healthcare, and this may affect the effectiveness of current vaccines and antibody therapies against SARS-CoV-2 infection. Like other coronaviruses, viral spike (S) glycoprotein that mediates cell entry is the primary target for neutralizing antibodies. The receptor-binding domain (RBD) on the trimeric S protein interacts with the virus receptor, angiotensin-converting enzyme (ACE2), therefore neutralizing the antibodies that effectively block RBD–ACE2 interaction, representing potential therapeutic options that could pave the way for the development of broad-spectrum therapeutics and vaccines against SARS-CoV-2 variants [1].

The recent circulating variants, namely B.1.1.7 (Alpha), B.1.351 (Beta), P.1 (Gamma), B.1.617.2 (Delta), and the most recent B.1.1.529 (Omicron) lineages, are variants of concern (VOC), according to the World Health Organization (WHO). These variants containing amino acid mutations within the S RBD, have shown an increased infectivity, and are associated with immune evasion from antibody-mediated protection [2]. Structural changes in the RBD are responsible for the enhanced or weakened binding of the virus to the cell receptor, which may affect the virus infectivity [3].

Furthermore, studies have also reported that certain neutralizing monoclonal antibodies (mAbs) developed for clinical use showed partial or complete loss of neutralization efficacy against newly emerged SARS-CoV-2 variants due to mutations at the antigenic supersite in the ACE2-receptor-binding motif (RBM) of the RBD, which is a major target of potent virus-neutralizing antibodies [4,5,6]. The RBM in the RBD comprises amino acids 438–506, which play a crucial role in determining affinity and directly interact with the ACE2 receptor [7]. It has been reported that several mutations in the SARS-CoV-2 RBM, such as N439K, L452R, S477N, T478K, E484K, S494P, N501Y, and A502S, have increased the infectivity and stability of SARS-CoV-2 [8]. Thus, there is an urgent need to identify and characterize the conserved epitopes in the RBM of S using broadly neutralizing mAbs, which will facilitate the development of potential therapeutics and the design of broadly protective vaccines against current and future VOC. The objective of this study was to identify and characterize the conserved epitopes in the SARS-CoV-2 RBD using broadly neutralizing antibodies against SARS-CoV-2 variants. 

## 2. Materials and Methods

### 2.1. Cell Line and Virus

Vero E6 cells (CRL-1586; ATCC) were grown in Dulbecco’s Modified Eagle’s Medium (DMEM, Gibco, Grand Island, NY, USA) and supplemented with 8% fetal bovine serum (FBS) and penicillin-streptomycin at 37 °C in 5% CO_2_. The SARS-CoV-2 B.1.1.7 (Alpha; GISASID Accession ID EPI_ISL_754083), B.1.351 (Beta; GISASID Accession ID EPI_ISL_1173248), and B.1.617.2 (Delta; Accession ID EPI_ISL_2621925) variants were obtained from the National Centre for Infectious Diseases, Singapore. Additionally, the SARS-CoV-2 wild-type (WT) strain (hCoV-19/Singapore/2/2020; GISASID Accession ID EPI_ISL_407987) was obtained from Duke-NUS Medical School, Singapore. 

### 2.2. Production of mAbs

BALB/c mice were immunized twice subcutaneously with a SARS-CoV-2 spike full-length protein (#SPN-C52H8, ACRObiosystems, Newark, DE, USA) emulsified with Freund’s adjuvant (Sigma-Aldrich, St. Louis, MO, USA). The mice were boosted with the spike RBD (Z03483, GenScript, Piscataway, NJ, USA) 3 days before the fusion of splenocytes with SP2/0 cells. The mAbs were generated as described previously [9]. Hybridomas were screened against SARS-CoV-2-infected Vero-E6 cells by immunofluorescence assay, as described below. The hybridomas that produced the mAbs and tested positive by indirect immunofluorescence assay were cloned by limiting dilution at least three times. The positive mAbs were then tested for authentic virus microneutralization against SARS-CoV-2 and surrogate virus neutralization against SARS-CoV-2 RBD, as described below.

### 2.3. Immunofluorescence Assay (IFA)

Vero E6 cells cultured in 96-well plates were infected with SARS-CoV-2 virus at a multiplicity of infection (MOI) of 0.1. At 36 h post-infection, the cells were fixed with 4% paraformaldehyde in phosphate-buffered saline (PBS) for 20 min at room temperature. The fixed cells were incubated with hybridoma culture supernatant for 1 h at room temperature, then incubated with a 1:100 dilution of fluorescein isothiocyanate (FITC)-conjugated rabbit anti-mouse immunoglobulin (Dako, Denmark) for 1 h at room temperature. The fluorescence signal was evaluated by wide-field epi-fluorescence microscopy (Olympus IX71, London, UK).

### 2.4. Authentic Virus Microneutralization (VMN) Activity of mAbs against SARS-CoV-2

To evaluate virus microneutralization activity of mAbs against the SARS-CoV-2 wild-type (WT) or Alpha (B.1.1.7), Beta (B.1.351), and Delta (B.1.617.2) variants, briefly, Vero E6 cells were seeded at 4 × 10^4^/well 5 h prior to infection in a 96-well plate. Then, quadruplicates of two-fold serially diluted mAbs in DMEM media supplemented with 2% FBS were incubated with 100 times of 50% tissue culture infectious doses (TCID_50_) of SARS-CoV-2 virus for 1 h. The virus–mAb mixture was added into wells containing a monolayer of Vero E6 cells and then incubated at 37 °C for 4 days. Cytopathic effect (CPE) was observed under an inverted microscope (Olympus IX71), and the neutralizing titer was assessed at the lowest concentration of mAb that provided complete protection. The half-maximal inhibitory concentration (IC_50_) values were calculated as the reciprocal concentration of mAb that achieved 50% inhibition of viral infection and were determined using a 4-parameter logistic regression curve by GraphPad Prism version 9 (GraphPad^®^ Software, San Diego, CA, USA).

### 2.5. SARS-CoV-2 Surrogate Virus Neutralization Test (sVNT)

To determine the neutralizing activity of mAbs against SARS-CoV-2 variants, a GenScript cPASS surrogate virus neutralization test (sVNT) was performed according to manufacturer’s instructions (GenScript, Piscataway, NJ, USA). Briefly, mAbs and negative and positive controls were incubated with horseradish peroxidase-conjugated SARS-CoV-2 WT RBD (HRP–RBD) or HRP–RBD variants (Table 1) such as Alpha RBD–HRP (B.1.1.7), Beta RBD–HRP (B.1.351), Gamma RBD–HRP (P.1), Kappa RBD–HRP (B.1.617.1), Delta RBD–HRP (B.1.617.2), Delta Plus/AY.1 RBD–HRP (B.1.617.2.1), or Epsilon RBD–HRP (B.1.429/7) in a 96-well plate for 30 min at 37 °C. Following incubation, the RBD–HRP and mAb mixtures were added onto a 96-well ELISA plate coated with human ACE2 protein and incubated for 30 min at 37 °C. The plates were then washed, removing RBD–HRP complexed with mAb. The reaction was developed with tetramethylbenzidine (TMB), followed by adding a stop solution. The optical density (OD) of each well was measured at 450 nm.

The percent inhibition of a mAb was calculated based on the OD intensity reduction caused by the mAb; the average OD of the negative control was used to calculate the percent inhibition. Percent inhibition = (1 − average OD of sample/average OD of negative control) × 100%. A sample with a percent inhibition ≥30% was considered positive [10].

### 2.6. Mapping of Neutralizing Epitopes in the Spike RBD by Escape Mutant Analysis Using 9G8 mAb

The epitope recognized by the highly neutralizing mAb 9G8 was mapped by characterization of escape mutants as described previously [11]. Briefly, SARS-CoV-2 at a MOI of 1 or 0.1 was incubated with different dilutions of mAb for 1 h at 37 °C. Next, the virus–mAb mixtures were incubated with Vero E6 cells in 24-well plates for 96 h at 37 °C. Virus replication was monitored, and the supernatant from the wells with evident viral replication was further passaged in the presence of mAb several times until a complete escape mutant was generated. The escape mutants were plaque purified for further characterization by sequencing, replication, and conducting VMN with mAb 9G8. The S gene was amplified and cloned into pJet 1.2 (CloneJET PCR cloning kit, Thermo Fisher Scientific, Waltham, MA, USA) for sequencing. The sequences of individual clones were analyzed by comparison with the sequences of the parent virus. 

### 2.7. Epitope Distribution Analysis

As of 28 November 2021, data on the analysis of 5,343,811 full-length SARS-CoV-2 spike sequences were made available at the GISAID CoVSurver (www.gisaid.org/epiflu-applications/covsurver-mutations-app, accessed on 28 November 2021) with the reference sequence ‘hCoV-19/Wuhan/WIV04/2019’ (EPI_ISL_402124). The neutralizing mAb 9G8 recognized amino acid residues at 483 and 489 and was evaluated for amino acid sequence variation compared to the reference sequence SARS-CoV-2/Wuhan/WIV04/2019. The total number of amino acid changes at each position was counted and converted to a frequency by dividing by the total number of sequences analyzed.

## 3. Results

### 3.1. Characterization of a Broadly Neutralizing Monoclonal Antibody against Spike-RBD

**Figure 1:**
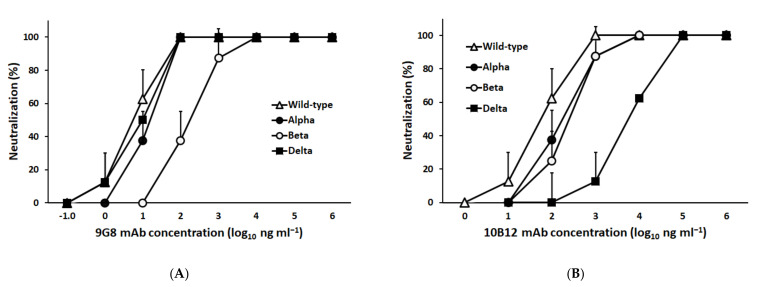
Neutralization of mAbs to authentic SARS-CoV-2 WT (hCoV-19/Singapore/2/2020), Alpha (B.1.1.7), Beta (B.1.351), and Delta (B.1.617.2) variants in Vero E6 cells. (**A**) The neutralizing potency of mAb 9G8 by virus microneutralization against 100 TCID_50_ of SARS-CoV-2 WT and variants. (**B**) The neutralizing potency of mAb 10B12 by virus microneutralization against 100 TCID_50_ of SARS-CoV-2 WT and variants. The half-maximal inhibitory concentration (IC_50_) values were determined using GraphPad Prism software (v. 9). The data presented are the mean of two biological replicates ± SD of the mean.

A panel of hybridomas secreting mAbs against S was screened for virus microneutralization against SARS-CoV-2, in addition to sVNT against SARS-CoV-2 RBD. Based on considerable binding efficacy with RBD, mAbs 9G8 and 10B12 belonging to IgM and IgG1 isotypes, respectively, were tested for cross-neutralizing potency against SARS-CoV-2 variants Alpha, Beta, and Delta by VMN and compared with SARS-CoV-2 WT. The results of the half-maximal inhibitory concentration (IC_50_) of mAb 9G8 showed 6.3 ng/mL, 10.2 ng/mL, and 10.5 ng/mL against SARS-CoV-2 WT, Delta, and Alpha, respectively (Figure 1A). However, mAb 9G8 showed an approximately 25-fold higher IC_50_ (158.2 ng/mL) against the SARS-CoV-2 Beta variant when compared to SARS-CoV-2 WT (Figure 1A). The IC_50_ of mAb 10B12 showed 62.9 ng/mL, 266.6 ng/mL, and 153.3 ng/mL against the SARS-CoV-2 WT, Beta, and Alpha variants, respectively; however, the mAb 10B12 did not neutralize the Delta variant well and showed more than a 100-fold higher IC_50_ (6.44 μg/mL) when compared to SARS-CoV-2 WT (Figure 1B).

**Figure 2:**
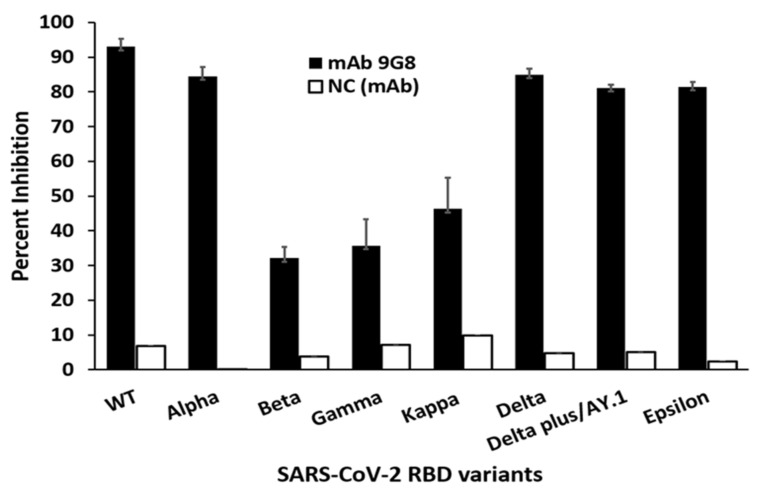
Analysis of mAb 9G8 blocking the SARS-CoV-2 spike RBD variant from binding to the human ACE2 receptor by surrogate virus neutralization test (sVNT). The percent inhibition of mAb 9G8 was measured against RBD of SARS-CoV-2 WT or SARS-CoV-2 variants such as Alpha (B.1.1.7), Beta (B.1.351), Gamma (P.1), Kappa (B.1.617.1), Delta (B.1.617.2), Delta Plus/AY.1 (B.1.617.2.1), and Epsilon (B.1.429/7). The mAb 6C8 specific for nucleocapsid protein was used as a negative control. The results were expressed as the arithmetic mean of percent-blocking values of triplicates and ±SD of the mean.

As mAb 9G8 neutralized all the tested SARS-CoV-2 variants, it was selected for further neutralization efficacy experiments against SARS-CoV-2 RBD variants. Given the difficulty of VMN assays against all the SARS-CoV-2 VOCs, we performed an sVNT using mAb 9G8 against RBD variants. Interestingly, the results showed that mAb 9G8 was able to inhibit more than 80% against the SARS-CoV-2 WT, Epsilon (B.1.429/7), Alpha (B.1.1.7), Delta (1.617.2), and Delta Plus (1.617.2.1) RBD variants (Figure 1B), whereas it showed moderate inhibition (46.3%) against the Kappa (B.1.617.1) RBD variant. Moreover, mAb 9G8 showed at least >30 percent inhibition towards the Gamma (P.1) (35.7%) and Beta (B.1.351) (32.1%) RBD variants (Figure 2).

### 3.2. Generation of In Vitro Escape Mutants against mAb 9G8

**Figure 3:**
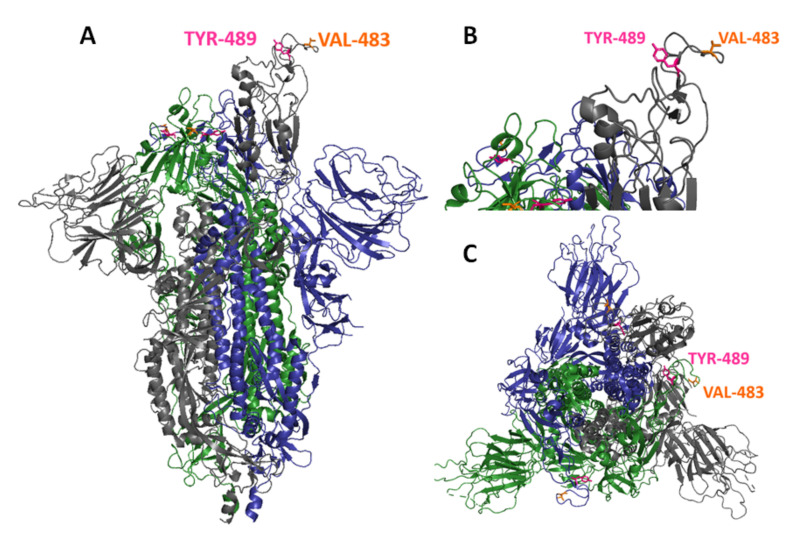
Antigenicity-mAb 9G8-related sites. Structural overview of SARS-CoV-2 S protein at open state based on PDB: 7DDN indicating the footprints of a broadly neutralizing antibody 9G8 on the RBD of S trimers. The S trimers are colored grey, blue, and green. The mAb binding amino acid residues Val 483 and Tyr 489 are indicated in orange and pink, respectively. (**A**) Upright view; (**B**) close-up view of SARS-CoV-2 S protein at open state; (**C**) top-down view.

The amino acid residues involved in forming the epitope of mAb 9G8 were analyzed using a selection of neutralizing escape mutants by SARS-CoV-2 viral infection in the presence of mAb 9G8. We generated escape mutants of SARS-CoV-2 WT passed in Vero-E6 cells eight times in the presence of mAb 9G8, and the escape mutants were plaque purified. The sequences of S from plaque-purified viruses carried single-point mutations at amino acid position V483F (Val to Phe) or Y489H (Tyr to His). Furthermore, based on the sequence analysis, purified virus plaque with a single amino acid mutation at Val 483 Phe (SARS-CoV-2WT/em483) or Tyr 489 His (SARS-CoV-2WT/em489) was assessed by virus neutralization assay. The results showed that any independent mutations at positions 483 and/or 489 of the parent viruses (Figure 3) caused a viral neutralizing evasion from mAb 9G8. Our study revealed that the amino acid residues V483F and Y489H within the RBD of an S protein might have an impact on ACE2 binding. The mutation frequency at these two amino acid positions in the RBD of SARS-CoV-2 S was compared against the SARS-CoV-2/Wuhan/WIV04/2019 reference S RBD sequence. Of the 5,343,811 available sequences, the mutation V483F appeared in 2699 sequences (0.0005%), and V483A appeared in 328 sequences, while 99.93% of the sequences were invariant. Moreover, amino acid residue at 489 showed more than 99.996% (5,343,664) sequences, which shows that they were highly conserved (as of 28 November 2021).

## 4. Discussion

The increasing number of new SARS-CoV-2 variants with multiple mutations in the RBD represents a great concern regarding the effectiveness of current vaccines and antibody-based therapeutics. The most recent VOC known as Omicron (B.1.1.529) was first identified in South Africa and contains thrice the number of S mutations as compared to the current dominant Delta variant [12]. Currently, preliminary findings show that the variant causes less severe disease compared to the Delta variant, but there has been evidence pointing to reduced efficacy of vaccines against the Omicron variant [13]. Thus, identification of highly conserved amino acid residues in the neutralizing epitopes of SARS-CoV-2 RBDs using broadly neutralizing monoclonal antibodies will be essential in designing comprehensive protective vaccines and therapeutics against current and future SARS-CoV-2 variants.

Initially, in this study, mAb 9G8 and 10B12 were selected based on their neutralizing ability against VMN and the sVNT assays against SARS-CoV-2 WT. After displaying a potent neutralizing efficacy against the Alpha, Beta, and Delta viruses in the VMN assays, mAb 9G8 was further selected for sVNT. Interestingly, it was able to neutralize all seven emerging SARS-CoV-2 variants tested, which included the Delta variant, the variant of greatest concern, which is now being replaced by the emerging Omicron variant. These results were consistent with the neutralizing efficacy of mAb 9G8 in the VMN assay.

The escape mutant analysis of mAb 9G8 proved Val 483 and Tyr 489 to be the amino acid residues in contact with the SARS-CoV-2 RBM region (Figure 3). Even though the mAb 9G8 recognizing amino acid residues were highly conserved among the RBD variants, it retained binding to most RBD variants when the mutations occurred in amino acid positions 417, 452, 478, and 501 of the RBD (Table 1). However, a reduced binding efficacy was observed against variants (Beta, Kappa, and Gamma) containing amino acid mutation at E484K or Q (Table 1). This might be due to the mutation located at amino acid residue 484, which is found in the RBD region of the Beta, Kappa, and Gamma variants [14]. Early research on the Omicron variant has shown multiple mutations in the RBM such as N440K, G446S, S477N, T478K, Q493K, G496S, Q498R, N501Y, Y505H, including a mutation residing at amino acid residue E484A [15], which is associated with reduced binding, as observed with the tested Beta, Kappa, and Gamma variants harboring the exact mutation. The amino acid residue V483 recognized by mAb 9G8 is adjacent to E484, which facilitates reduction in inhibition against the Beta, Kappa, and Gamma variants. V483 serves as a critical amino acid residue in the RBM of the RBD because it is in close proximity to Q24 of ACE2, one of the interacting amino acid residues of ACE2 [16]. Studies have reported that the mutation at position V483 is located at the RBM of the S1 protein, which has the ability to change the protein’s secondary structure, making the mutation unique [17]. A study has also indicated that mutations at amino acid residues including 483 become resistant to some neutralizing antibodies and convalescent sera [18]. In addition, mAb 9G8 recognizes one of the invariant amino acid residues, Y489, localized within the tip of the RBD, where it interacts with amino acid residue M82 of ACE2 [19]. A recent study using a computational approach reported that variants with mutations involving Y489 in the RBD are prone to destabilize the binding after single amino acid replacements [20]. Additionally, the amino acid Y489 has also been found to be a hotspot that disrupts the binding of RBM to neutralizing antibodies as predicted [20]. The amino acid mutation Y489H has occurred only 46 times in 15 countries and is a highly conserved amino acid residue in the RBM of the RBD (as of 28 November 2021). The possible limitation in our study was the continuous evaluation of new variants of SARS-CoV-2 with multiple mutations in the RBD that have a probability to reduce the potency of neutralizing monoclonal antibodies, including mAb 9G8. Therefore, the inclusion of mAb 9G8 in combination with other neutralizing mAbs targeting conserved regions can offer cross-protection against current and future VOCs.

In conclusion, the antigenic mapping of RBDs using highly cross-reactive neutralizing mAb 9G8 revealed that the amino acid residues at position 483 and 489 in the RBM might also be the key residues responsible in the interaction of both their common functional receptor, ACE2, and neutralizing antibodies. Antibodies that target conserved epitopes in the RBD will provide more information on the development of broadly protective therapeutics and vaccine designs against potential variants of SARS-CoV-2.

## Figures and Tables

**Table 1:** SARS-CoV-2 variants and their RBD mutations used in this study.

SARS-CoV-2 Variant	Lineage	RBD Mutations
L	Wuhan-Hu-1	N/A
Alpha	B.1.1.7	N501Y
Beta	B.1.351	K417N, E484K, N501Y
Gamma	P.1	K417T, E484K, N501Y
Kappa	B.1.617.1	L452R, E484Q
Delta	B.1.617.2	L452R, T478K
Delta Plus/AY.1	B.1.617.2.1	K417T, L452R, T478K
Epsilon	B.1.429	L452R

RBD, SARS-CoV-2 receptor-binding domain; N/A, not applicable.

## References

[1] Hoffmann M., Arora P., Groß R., Seidel A., Hörnich B.F., Hahn A.S., Krüger N., Graichen L., Hofmann-Winkler H., Kempf A. (2021). SARS-CoV-2 variants B.1.351 and P.1 escape from neutralizing antibodies. Cell.

[2] Wibmer C.K., Ayres F., Hermanus T., Madzivhandila M., Kgagudi P., Oosthuysen B., Lambson B.E., de Oliveira T., Vermeulen M., van der Berg K. (2021). SARS-CoV-2 501Y.V2 escapes neutralization by South African COVID-19 donor plasma. Nat. Med..

[3] Lan J., Ge J., Yu J., Shan S., Zhou H., Fan S., Zhang Q., Shi X., Wang Q., Zhang L. (2020). Structure of the SARS-CoV-2 spike receptor-binding domain bound to the ACE2 receptor. Nature.

[4] Wang P., Nair M.S., Liu L., Iketani S., Luo Y., Guo Y., Wang M., Yu J., Zhang B., Kwong P.D. (2021). Antibody resistance of SARS-CoV-2 variants B.1.351 and B.1.1.7. Nature.

[5] Chen R.E., Zhang X., Case J.B., Winkler E.S., Liu Y., VanBlargan L.A., Liu J., Errico J.M., Xie X., Suryadevara N. (2021). Resistance of SARS-CoV-2 variants to neutralization by monoclonal and serum-derived polyclonal antibodies. Nat. Med..

[6] Planas D., Veyer D., Baidaliuk A., Staropoli I., Guivel-Benhassine F., Rajah M.M., Planchais C., Porrot F., Robillard N., Puech J. (2021). Reduced sensitivity of SARS-CoV-2 variant Delta to antibody neutralization. Nature.

[7] Du Y., Wang H., Chen L., Fang Q., Zhang B., Jiang L., Wu Z., Yang Y., Zhou Y., Chen B. (2021). Non-RBM Mutations Impaired SARS-CoV-2 Spike Protein Regulated to the ACE2 Receptor Based on Molecular Dynamic Simulation. Front. Mol. Biosci..

[8] Plante J.A., Mitchell B.M., Plante K.S., Debbink K., Weaver S.C., Menachery V.D. (2021). The variant gambit: COVID-19’s next move. Cell Host Microbe.

[9] Yokoyama W.M., Christensen M., Santos G.D., Miller D. (2013). Production of monoclonal antibodies. Curr. Protoc. Immunol..

[10] Tan C.W., Chia W.N., Young B.E., Zhu F., Lim B.L., Sia W.R., Thein T.L., Chen M.I., Leo Y.S., Lye D.C. (2021). Pan-Sarbecovirus Neutralizing Antibodies in BNT162b2-Immunized SARS-CoV-1 Survivors. N. Engl. J. Med..

[11] Kaverin N.V., Rudneva I.A., Ilyushina N.A., Varich N.L., Lipatov A.S., Smirnov Y.A., Govorkova E.A., Gitelman A.K., Lvov D.K., Webster R.G. (2002). Structure of antigenic sites on the haemagglutinin molecule of H5 avian influenza virus and phenotypic variation of escape mutants. J. Gen. Virol..

[12] Callaway E. (2021). Heavily mutated Omicron variant puts scientists on alert. Nature.

[13] Sheikh A., Kerr S., Woolhouse M., McMenamin J., Robertson C. (2021). Severity of Omicron Variant of Concern and Vaccine Effectiveness against Symptomatic Disease: National Cohort with Nested Test Negative Design Study in Scotland. Edinb. Res. Explor..

[14] Koehler M., Ray A., Moreira R.A., Juniku B., Poma A.B., Alsteens D. (2021). Molecular insights into receptor binding energetics and neutralization of SARS-CoV-2 variants. Nat. Commun..

[15] Shah M., Woo H.G. (2021). Omicron: A heavily mutated SARS-CoV-2 variant exhibits stronger binding to ACE2 and potently escape approved COVID-19 therapeutic antibodies. bioRxiv.

[16] Ashwaq O., Manickavasagam P., Haque S.M. (2021). V483A: An emerging mutation hotspot of SARS-CoV-2. Future Virol..

[17] Nguyen T.T., Pathirana P.N., Nguyen T., Nguyen Q.V.H., Bhatti A., Nguyen D.C., Nguyen D.T., Nguyen N.D., Creighton D., Abdelrazek M. (2021). Genomic mutations and changes in protein secondary structure and solvent accessibility of SARS-CoV-2 (COVID-19 virus). Sci. Rep..

[18] Li Q., Wu J., Nie J., Zhang L., Hao H., Liu S., Zhao C., Zhang Q., Liu H., Nie L. (2020). The Impact of Mutations in SARS-CoV-2 Spike on Viral Infectivity and Antigenicity. Cell.

[19] Adams L.E., Dinnon K.H., Hou Y.J., Sheahan T.P., Heise M.T., Baric R.S. (2021). Critical ACE2 Determinants of SARS-CoV-2 and Group 2B Coronavirus Infection and Replication. mBio.

[20] Tsai K.C., Lee Y.C., Tseng T.S. (2021). Comprehensive Deep Mutational Scanning Reveals the Immune-Escaping Hotspots of SARS-CoV-2 Receptor-Binding Domain Targeting Neutralizing Antibodies. Front. Microbiol..

